# Single-Cell RNA Sequencing Reveals Distinct Cardiac-Derived Stromal Cell Subpopulations

**DOI:** 10.3390/jcdd9110374

**Published:** 2022-11-01

**Authors:** Jessica R. Hoffman, Arun R. Jayaraman, Sruti Bheri, Michael E. Davis

**Affiliations:** 1 Wallace H. Coulter Department of Biomedical Engineering, Georgia Institute of Technology & Emory University School of Medicine, Atlanta, GA 30322, USA; 2 Molecular & Systems Pharmacology Graduate Training Program, Graduate Division of Biological & Biomedical Sciences, Laney Graduate School, Emory University, Atlanta, GA 30322, USA; 3 Children’s Heart Research & Outcomes (HeRO) Center, Children’s Healthcare of Atlanta & Emory University, Atlanta, GA 30322, USA

**Keywords:** heart failure, single cell RNA sequencing, c-kit+ cardiac stromal cells

## Abstract

Human cardiac-derived c-kit+ stromal cells (CSCs) have demonstrated efficacy in preclinical trials for the treatment of heart failure and myocardial dysfunction. Unfortunately, large variability in patient outcomes and cell populations remains a problem. Previous research has demonstrated that the reparative capacity of CSCs may be linked to the age of the cells: CSCs derived from neonate patients increase cardiac function and reduce fibrosis. However, age-dependent differences between CSC populations have primarily been explored with bulk sequencing methods. In this work, we hypothesized that differences in CSC populations and subsequent cell therapy outcomes may arise from differing cell subtypes within donor CSC samples. We performed single-cell RNA sequencing on four neonatal CSC (nCSC) and five child CSC (cCSC) samples. Subcluster analysis revealed cCSC-enriched clusters upregulated in several fibrosis- and immune response-related genes. Module-based analysis identified upregulation of chemotaxis and ribosomal activity-related genes in nCSCs and upregulation of immune response and fiber synthesis genes in cCSCs. Further, we identified versican and integrin alpha 2 as potential markers for a fibrotic cell subtype. By investigating differences in patient-derived CSC populations at the single-cell level, this research aims to identify and characterize CSC subtypes to better optimize CSC-based therapy and improve patient outcomes.

## 1. Introduction

Cell therapy has emerged as a promising therapeutic strategy for the treatment of diseases, including auto-immune disease, blood disorders, cancer, neurodegenerative disease, and cardiovascular disease [1,2]. Various tissue-specific cells, blood cells, and stem cells have been clinically approved, including the use of autologous mesenchymal stem cells for acute myocardial infarction. Unfortunately, cell therapy has been hampered by mixed results, in part due to high cell heterogeneity. Unlike small molecule drugs, cells are highly variable, adaptive to biological cues, and complex in their mechanisms of action. Inconsistencies in cell therapy trials may be explained by batch-to-batch or patient-to-patient variation. Specifically, cell donor age and disease have been shown to negatively impact cell efficacy, reducing the effectiveness of cardiac-derived progenitor cells [3,4,5], adipose stem cells [6,7], and mesenchymal stem cells [8,9,10,11], among others [12]. Given the high heterogeneity of cell populations, emphasis has been placed on identifying mechanisms of repair and markers of “good” cells. Recent studies have leveraged single-cell RNA sequencing to identify subpopulations of cells that may be driving therapeutic efficacy [13,14,15]. By identifying potential cell surface markers of reparative cells, researchers will be able to isolate and/or enrich for optimal cell populations.

Currently, autologous human cardiac-derived c-kit+ stromal cells (CSCs) are under investigation in the CHILD clinical trial for the treatment of hypoplastic left heart syndrome, a complex congenital heart disease (NCT03406884) [16]. Preclinical results indicate CSCs induce repair in damaged myocardium [4,5,17,18]. While there is clear evidence that these cells do not become cardiomyocytes and their use was considered controversial, the reparative potential of pediatric CSCs focuses on the paracrine effects of these cells. Recent results from the phase II CONCERT-HF trial (NCT02501811) suggest that a combination of CSCs and MSCs improves clinical outcomes in patients with ischemic heart failure [19]. Previous research investigating CSC heterogeneity has demonstrated that cell culture conditions (e.g., hypoxia [20,21,22] and cell aggregation [23]), as well as donor age [4,24] and disease status, affect CSC composition and therapeutic potential. Additionally, CSC reparative outcomes may be linked to age: CSCs derived from neonate patients (nCSCs) outperform cells derived from older patients [3,4,22,24,25,26]. Specifically, nCSCs possess greater anti-fibrotic signaling, reduced immune response, and increased chemotaxis capabilities in comparison to child CSCs (cCSCs) [4].

Nevertheless, differences among CSC populations have been primarily investigated using bulk sequencing methods, which treat patient-derived cells as a homogenous sample. Here, we hypothesized that variance in patient outcomes may be driven by differences in cell subtypes or subpopulations and that CSCs transition to reduced reparative states as patients age. To address this hypothesis, we used single-cell RNA sequencing to (1) identify potentially phenotypically different cell subpopulations and (2) map transcriptomic trajectories of cells from CSCs of the neonate (*n* = 4) and child (*n* = 5) congenital heart disease patients. Overall, we uncovered a more heterogenous cell population among older patient samples and identified fibrotic and inflammatory cell subpopulations within these samples, which may explain differences in therapeutic outcomes. Our trajectory and differential expression analyses unveiled differences between cells belonging to a fibrotic cell cluster and cells belonging to clusters enriched in cell cycle and cell proliferation processes. We identified markers of this fibrotic cell cluster—versican (VCAN) and integrin subunit alpha 2 (ITGA2)—isolated these cells using fluorescence-activated cell sorting and observed lower cell proliferation in this subpopulation. Ultimately, by identifying and distinguishing pro- and non-reparative CSC populations, it may be possible to improve cell therapy outcomes.

## 2. Materials Methods

### 2.1. CSC Culture and Expansion

Cells collected from the right atrial appendage of five neonatal (<1 month) and five child (3.43 years ± 2.6 years) patients with congenital heart disease were separated for c-kit+ CSCs using magnetic cell sorting. Patient characteristics for samples used in the study are listed in Table S1. Cells were cultured in Ham’s F-12 medium (Corning Cellgro®, Corning, NY, USA) with 10% fetal bovine serum, 1% penicillin-streptomycin, 1% l-glutamine, and 0.04% human fibroblast growth factor-β. Characterization of CSC populations was analyzed using an Aurora Flow Cytometer (Cytek) (Figure S1). Sorted cells were expanded in culture and submitted for single-cell RNA sequencing between passages 5 and 15. Cells were washed with PBS 2× before sequencing to reduce the presence of ambient RNA and help ensure a high quality of RNA. Sequencing was performed at the Emory University NPRC Genomics Core at a depth of 50,000 reads/cell (10× Genomics Chromium Controller). The 10× system used Next GEM technology to perform droplet-based cell capture and barcoding. Total reads and other sequencing metrics are listed in Table S2.

### 2.2. Computational Methods

Raw reads from single-cell sequencing were processed using CellRanger (10× Genomics, v6.0.0) [27]. Quality control metrics are listed in Table S2. Data are available at Gene Expression Omnibus accession # GSE204928. The doublets were filtered using Scrublet and Scanpy, and the raw count’s data was processed using the Seurat package in R [28,29,30]. Cells with 1000–7000 distinctly expressed genes and mitochondrial gene fraction totaling < 5% of total transcript counts were kept. One neonatal patient sample (Patient 985) was removed due to low transcript counts (<4000) and a small number of distinctly expressed genes (<2000). A total of 72,798 cells were sequenced, and 52,293 cells were analyzed after filtering (Table S2).

Data from patient samples were integrated by first normalizing counts using the SCTransform method with variation due to mitochondrial gene fraction regressed out of the datasets [31]. The patient datasets were combined using the comprehensive integration methodology implemented in Seurat: integration features and anchors were selected using default parameters. Integrated data were then scaled, and principal component analysis (PCA) and uniform manifold approximation and projection (UMAP) were performed. Thirty principal components were considered. Thirteen cell clusters were identified using the Louvain community finding algorithm. Differential expression was computed on non-batch corrected data using the FindAllMarkers function and the Wilcoxon rank sum method. Putative cell type identities for each cluster were estimated using the SCType scoring algorithm (Table S3) [32]. Scores were computed for each cell cluster using a curated list of heart tissue cell gene markers (Table S3).

**Figure 1:**
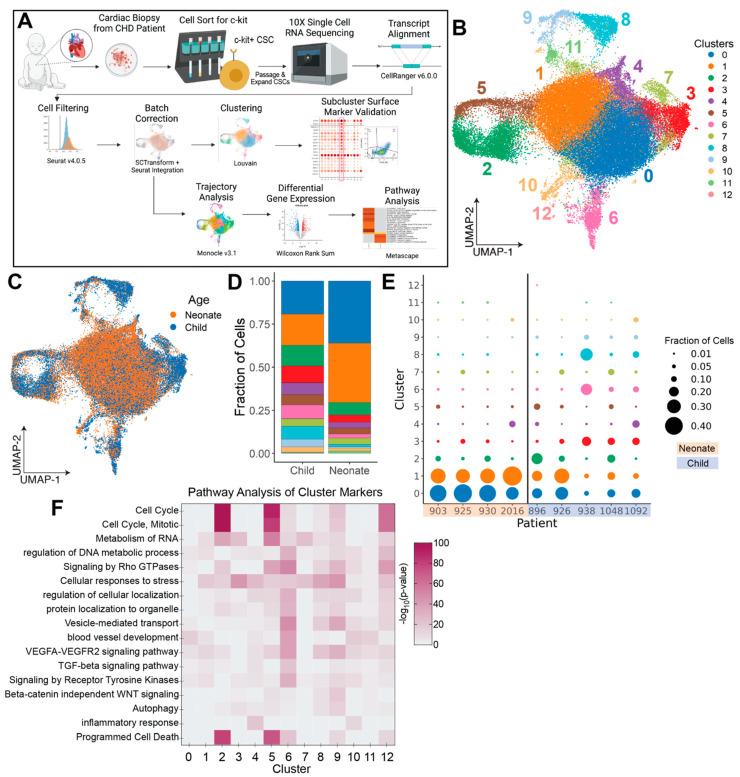
Clustering and cluster compositions of nCSCs and cCSCs. (**A**) Top sequence: CSC isolation from CHD patients and culture. Bottom sequence: analysis pipeline and computational tool summary. Figure generated in BioRender. UMAP projections of all patient-derived CSCs colored by (**B**) cell cluster and (**C**) age group. Cluster composition as grouped by (**D**) age group and (**E**) patient sample. (**F**) Enrichment of selected pathways from positive cluster marker gene sets, as listed in Table S6.

Trajectories were constructed using Monocle 3 with the “ncenter” parameter in the learn_graph function set to 500. Pseudotimes were computed by setting the root node as the cluster of interest and allowing the monocle to compute pseudotime values for the remaining cells. The dataset was batch corrected using the Batchelor alignment methodology implemented in Monocle. Co-expression of genes was computed along trajectories using the Moran’s I statistic as implemented in Monocle, and highly co-expressed genes with a q-value < 0.05 were clustered into 21 gene modules using the Leiden community detection algorithm (Table S4) [33,34,35,36,37,38]. A summary of the analysis pipeline is shown in Figure 1A.

Principal component analysis of samples was performed with pseudobulk data (“AggregateExpression” Seurat function, Figure S2). Differential expression analysis comparing nCSCs and cCSCs within each cell cluster was performed using edgeR (Figure S2). Gene expression was first aggregated by taking the sum of cell counts for each gene. Lowly expressed genes were filtered out using edgeR’s “filterByExpr” function with default parameters. A differential expression model was constructed using cell passage and age group co-variates (glmFit, Benjamini Hochberg correction). Differentially expressed genes were considered (FDR < 0.05, log2FC > 1, Table S5).

Surface proteins were identified using the cell surface protein atlas validated surface proteomes dataset [39]. The surface proteome dataset was filtered for proteins for which there was high confidence of expression on the cell surface. The dataset was also further filtered for the cluster of differentiation (CD) proteins for better identification of cell surface proteins. The dataset was analyzed for differentially expressed genes that are conserved across donor samples within the same cluster. The differentially expressed genes were then filtered for only genes present in the filtered surface proteome dataset for the determination of the highest transcriptionally expressing surface proteins.

### 2.3. Cell Sorting of CSC Subpopulations

Fluorescence-activated cell sorting was utilized for the isolation of CSC subpopulations based on the expression of the versican and integrin alpha 2 surface proteins. Anti-versican (Creative Biolabs, CBMAB-C9301-LY) and anti-integrin alpha 2 (R&D Systems, FAB1233P) antibodies conjugated to Alexa Fluor 647 and PE, respectively, were selected for analysis. Zombie Yellow™ dye (Biolegend) was used to assess cell viability. CSCs from patient 926, as well as pooled child CSCs from patients 926, 938, and 902 (4-year-old patient with atrial septal defect), were stained using manufacturers’ suggested concentration. Samples were sorted using a Sony SH800 Self Run Cell Sorter (Sony Biotechnology).

## 3. Results

### 3.1. Clustering and Compositional Analysis Reveal Differences in Neonate and Child CSCs

To identify cell subtypes in patient-derived CSC samples, we performed initial cell clustering with Louvain to identify thirteen CSC subpopulations (Figure 1B,C). Neonate-derived samples were largely enriched in clusters 0 and 1, while child-derived samples were enriched in clusters 3, 6, 8, and 9 (Figure 1D,E). Notably, we observed a higher level of sample-to-sample variability in child-derived samples. Patients 896 and 926 possessed a more neonate-like clustering profile, whereas patients 938, 1048, and 1092 produced a more dissimilar clustering profile with fewer cells represented in clusters 0 and 1 and more cells represented in clusters 3, 6, 8, and 9 (Figure 1E). Next, we identified positive cluster markers and performed pathway analysis to determine the biological significance of these gene sets. Markers for each cluster and pathway analysis results are listed in Tables S6 and S7, respectively. Our analyses indicated that cCSC-enriched cluster 6 is related to TGF-β and general receptor tyrosine signaling pathways (Figure 1F). Further, while fewer positive markers were identified for nCSC-enriched clusters 0 and 1, top biological pathways from these gene lists included extracellular matrix organization, blood vessel development, and cytoplasmic translation (Table S7). Finally, clusters 2 and 5 (representative of both nCSCs and cCSCs) were highly enriched in cell cycle processes, programmed cell death, and RNA metabolism.

### 3.2. Trajectory Analysis Identifies Co-Expressed Genes within CSC Subpopulations

**Figure 2:**
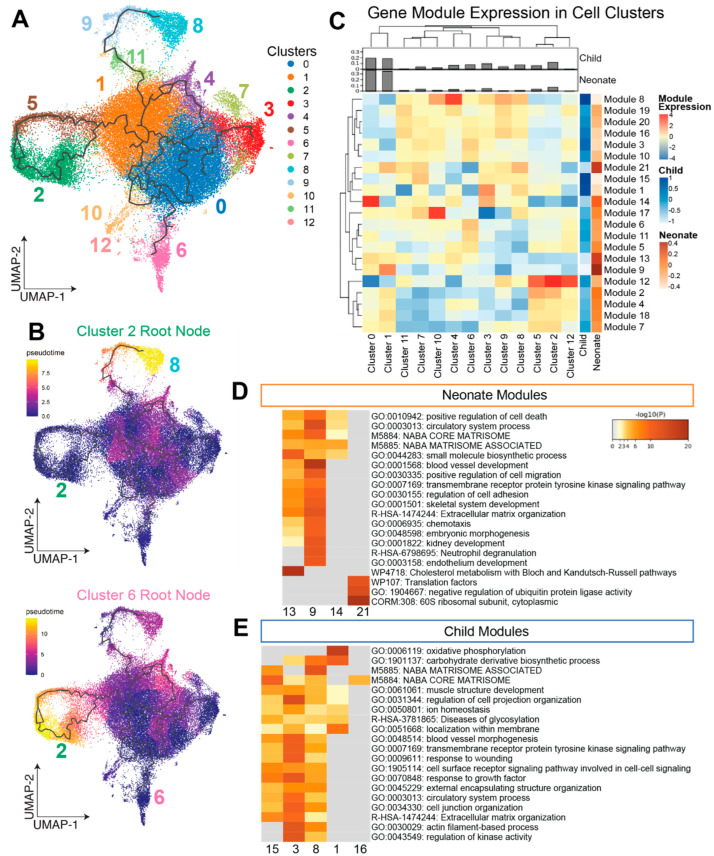
Trajectory analysis and gene clustering. UMAP projections with trajectories determined by Monocle colored by (**A**) monocle clusters and (**B**) pseudotime with root nodes set to cluster 2 (top) and cluster 6 (bottom) from the Seurat analysis. (**C**) gene module expression heatmap by Seurat cluster. Modules were determined through a Leiden clustering of highly co-expressed genes along trajectories. Module expression levels are computed by age group and are shown in the two rightmost columns in the heatmap. Cluster proportions by age group are illustrated by bar charts on top of the heatmap. Pathway analysis for the highest expressing modules among (**D**) neonates and (**E**) children highlights important biological differences between the age groups.

To understand how transcriptomic profiles change as cells move between CSC subpopulations, we performed trajectory analysis with Monocle 3 (Figure 2A). We computed pseudotimes using various clusters as the starting or root node. Notably, pseudotimes computed using cluster 2 cells (enriched in proliferative and cell cycle processes) as the root node resulted in the highest pseudotimes in cluster 8 cells (enriched in processes associated with oxidative stress and stimuli), indicating the transcriptomic profiles of these cells to be the most distinct from the cluster 2 cells (Figure 2B). Alternatively, pseudotimes computed using cluster 6 (enriched in fiber organization) as the root node resulted in the largest pseudotimes at cluster 2 (Figure 2B).

Next, to relate the previously determined cell clusters to gene sets, we computed co-expressed gene modules from our trajectory analysis. Co-expression of genes was computed along trajectories categorized based on Moran’s I statistic computed in Monocle, where a higher value indicates a higher level of co-expression with cells in similar positions of the trajectory. Highly co-expressed genes were clustered using the Leiden algorithm into 21 gene modules (Figure 2C, Table S4). Some modules corresponded strongly with certain cell clusters from the Seurat analysis. For example, cluster 4 cells had high expression of genes in module 8, while cluster 2 and 5 cells had high expression of module 12 genes.

Relating gene modules to CSC age groups, we determined that nCSCs were highly upregulated in genes belonging to modules 9, 13, 14, and 21, and cCSCs were upregulated in genes belonging to modules 1, 3, 8, 15, and 16. Pathway analysis of module 13 and 21 genes (upregulated in nCSCs) indicates enrichment of pathways related to small molecule biosynthesis and ribosomal activity, respectively (Figure 2D,E). Module 8 genes (upregulated in cCSCs) contained several immune-related cytokines, including *IL6* and *IL1B*. Module 9 contained notable gene members *CD34* and *PDGFB* and contained genes associated with chemotaxis. Across both age groups, modules 3, 9, 13, and 15 contained genes linked to extracellular matrix organization. 

### 3.3. cCSC-Enriched Cell Clusters 4 and 6 Are Upregulated in Inflammatory Cytokines and Fibrosis-Associated Genes

**Figure 3:**
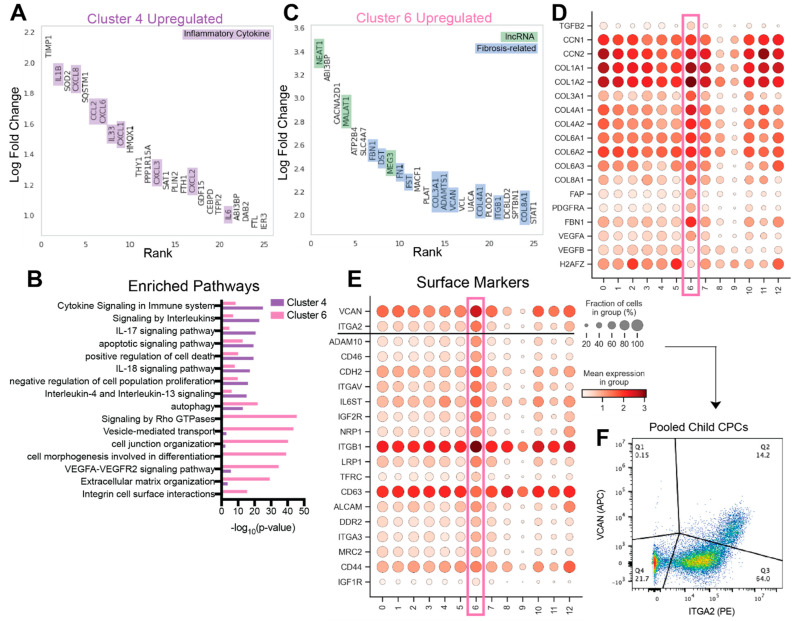
Characterization of inflammatory and fibrotic cell clusters 4 and 6. (**A**) Top 25 differentially expressed genes ordered by log fold change between cluster 4 cells and non-cluster 4 cells. Inflammatory cytokines are highlighted in purple. (**B**) Pathway analysis barplot of upregulated differentially expressed genes for clusters 4 and 6 cells. (**C**) Top 25 differentially expressed genes ordered by log fold change between cluster 6 cells and non-cluster 6 cells. Long non-coding RNAs (lncRNAs) are highlighted in green and fibrosis and extracellular matrix-related RNAs are highlighted in blue. (**D**) Dot plot of selected genes relating to fibrosis, angiogenesis, and proliferation. (**E**) Transcriptional expression of conserved differentially expressed surface proteins in cluster 6 cells. (**F**) Identification of a cluster 6-like population of interest in pooled child CSCs: ITGA2+, VCAN+.

To understand transcriptional differences between CSC subpopulations and identify genes that mark non-reparative cells, we examined differential gene expression in cCSC-enriched cell clusters. Notably, differential gene expression analysis identified several cytokines upregulated in cluster 4 cells, such as *IL1β*, *CXCL8*, *CCL2*, *CXCL6*, *IL33*, *CXCL1-3*, and *IL6* (Figure 3A). Pathway analysis indicated the enrichment of immune-related signaling pathways, including genes involved in the IL-17 and IL-18 signaling pathways (Figure 3B). In addition, cluster 4 was enriched in apoptotic signaling and negative regulation of cell proliferation processes. Many of the differentially expressed genes from this cluster were captured by the module 8 gene cluster, potentially indicating many of the cytokines expressed by these cells are driven by similar biological processes. This cell subpopulation was enriched in cCSCs; however, analysis of donor-specific clustering profiles indicates one nCSC sample (Patient 2016) had a high proportion of these cells (Figure 1E).

Next, we identified several fibrosis-related genes in cCSC-enriched cluster 6. Pathway analysis demonstrated enrichment of extracellular matrix organization and integrin cell surface interactions (Figure 3B), as well as TGF-β signaling (Figure 1B). Specifically, this cell population displayed high expression of several different types of collagen and genes associated with fibrosis, including *TGFB2*, *CCN1*, *CCN2*, and *FBN1* (Figure 3C,D) [40,41]. *PDGFRA* and *FAP,* known fibroblast markers that correlate with an epithelial-to-mesenchymal transition, and myofibroblast markers POSTN and PLOD2 were also upregulated in cluster 6 [42,43]. Further, well-studied long non-coding RNAs *NEAT1*, *MEG3*, and *MALAT1* are among the most upregulated RNAs in cell cluster 6 and have been shown to contribute to myocardial injury and adverse remodeling (Figure 3C). Multiple groups have determined that MALAT1 and NEAT1 play a role in cardiac fibrosis [44,45], and MEG3 has been shown to promote myocardial damage in an ischemia-reperfusion model by enhancing myocyte apoptosis and decreasing cell proliferation [46]. Cluster 6 cells showed high expression of fibronectin, a critical player during cardiac repair [47]. Finally, cluster 6 is also upregulated in angiogenic markers such as *VEGFA* and downregulation of the proliferation-related gene *H2AFZ*. Overall, cluster 6 may represent a pro-fibrotic, anti-proliferative subpopulation, potentially contributing to less reparative outcomes from older CSCs.

### 3.4. Identification of Non-Reparative Surface Markers for Cell Sorting

To identify markers and isolate less-desirable, pro-fibrotic cluster 6 cells, we examined differentially expressed surface markers using the cell surface protein atlas database (Figure 3E) [39]. Primary anti-versican and anti-ITGA2 antibodies were selected to characterize and sort patient 926 cCSCs and pooled cCSC subpopulations. Child CSCs were pooled previously and were comprised of patients 926, 938, and 902 (a 4-year-old patient with atrial septal defect). Cell viability was confirmed with a Zombie Yellow™ dye (>85% viability, data not shown). A subpopulation of cells with high versican and ITGA2 expression make up approximately 14% of the pooled child CSCs (Figure 3F). Our trajectory analysis results showed a marked difference between cluster 6 cells and cluster 2 cells enriched in cell cycle-related genes (Figure 2B). Therefore, we measured cell proliferation of unsorted nCSCs, cCSCs, and VCAN+/ITGA2+ cCSCs. Our results suggest that VCAN+/ITGA2+ cCSCs may represent a less proliferative cell population (Figure S3).

## 4. Discussion

There are clear age-dependent therapeutic differences in neonate and child CSC populations [3,4]. In comparison to cCSCs, nCSCs demonstrate greater anti-fibrotic potential, cell proliferation and chemotaxis, and enhanced secretion of cardioprotective paracrine factors [4]. Importantly, previous studies have isolated CSCs from patient cardiac biopsies using c-kit+ selection and explored age-dependent differences between nCSCs and cCSCs using bulk RNA sequencing and arrays [3,4,48]. This approach, however, masks the identity of potential cell subpopulations and attributes sample variance to patient variables. Here, we aimed to understand how these macroscopic dynamics present at the single-cell level, and whether we would be able to discern CSC subpopulations for selection, or depletion, with cell surface markers. To do so, we computed initial cell clusters—our CSC subpopulations. Then, to determine the major differences between cell clusters, we examined both differentially expressed genes and the enrichment of co-expressed gene modules. By combining multiple single-cell analysis methods, we uncovered potential phenotypes of CSC subpopulations that may explain CSC variability.

First, we expected to find major differences in nCSCs and cCSCs. Indeed, nCSCs largely clustered among clusters 0–5, whereas a considerable portion of cCSCs clustered in the offshoot branches of the UMAP projection, namely clusters 3, 4, 6, 8, and 9 (Figure 1B–E). Furthermore, given the previously demonstrated reduced performance of cCSCs, we hypothesized that cCSC samples are more heterogenous and may represent cells transitioning to a less reparative state. Here, we identified a high level of sample-to-sample variability among child patients, with some samples having more neonate-like clustering profiles than others (Figure 1E). Most obviously, the two cCSC samples with neonate-like clustering profiles also corresponded to the youngest of the child patient cohort (Patients 896 and 926, 12 months and 14 months old). Based on this observation, we ran a quasi-Poisson regression model to assess gene expression variability dependent on patient age. Ultimately, these results mirrored the results from the clustering-based analysis (Figure S4; Table S8). Genes such as *ABI3BP* and *CXCL6* that were upregulated in cCSC-enriched clusters also expressed highly in older patients, while genes such as *CXCL12* and *CXCR4* that are upregulated in nCSC-enriched clusters had higher expression in younger patients.

Interestingly, we found evidence for the enrichment of pro-inflammatory cell subpopulations and gene modules in cCSC samples, as compared to nCSC samples. First, cluster 4 cells showed high expression of several inflammation- and immune-related cytokines, including *IL1β*, *CXCL8,* and *IL6* (Figure 3A). While some CSCs from neonate patients 2016 were found in this cluster, cluster 4 was overall enriched in cCSCs (Figure 1E). Second, we identified age-related differences among the composition of cytokine gene modules 8 and 9, determined with trajectory analysis. nCSC-enriched module 9 cytokines were more strongly associated with chemotaxis, whereas cCSC-enriched module 8 included inflammatory-related cytokines (Figure 2C–E). Furthermore, we found a high positive correlation between cluster 4 and gene module 8. These results contradict a recent study by Vagnozzi et al., which challenged the efficacy of CSCs, attributing reparative function to an acute inflammatory-based wound healing response after cell delivery [49]. Of note, this work was completed in a mouse model of ischemia-reperfusion injury with murine CSCs, and CSC efficacy was evaluated two weeks after injection. Nevertheless, other studies investigating human CSCs from neonate patients corroborate our single-cell results. The results reported here are consistent with our previous research indicating that cCSCs drive an increased immune response and nCSCs induce higher levels of mesenchymal stem cell chemotaxis [4]. Pathway analysis of gene array data comparing nCSCs and cCSCs demonstrated enrichment of anti-inflammatory response in nCSCs [4]. Additionally, in a rat model of myocardial infarction, nCSCs reduced macrophage infiltration in the myocardium post-injury compared to adult CSCs [5].

Another cCSC-enriched subpopulation, cluster 6, showed high expression of genes related to fibrosis and angiogenesis, including *ITGB1*, *FBN1*, *DST*, *FN1*, *FST*, *ADAMTS1*, and *COL3A1*/*4A1*/*8A1*(Figure 3B,C). Given the strong connection of cluster 6 to adverse remodeling processes and the upregulation of fibrotic genes, we sought to identify markers for this cell subpopulation. We identified *ITGA2* and *VCAN*, a proteoglycan extracellular matrix regulator, as candidate markers for cluster 6 cells (Figure 3E). We used fluorescence-activated cell sorting to confirm surface protein expression and sort for a VCAN+/ITGA2+ subpopulation with pooled cCSCs and patient 926 cCSCs (Figure 3F). We measured cell proliferation and observed a trend for lower proliferation in the VCAN+/ITGA2+ cCSC subpopulation, as compared to the unsorted cCSC and nCSC populations (Figure S3). These results support our trajectory analysis, indicating that cluster 6 and cluster 2 (cell-cycle process enriched cell cluster) are the most dissimilar. Future studies should confirm this subpopulation’s pro-fibrotic function and sort out these deleterious cell subtypes to enhance CSC treatment efficacy.

Furthermore, we assigned putative cell labels to our CSC subpopulations to understand which cell types may be present in our samples (Table S3). Our results indicate that clusters 0, 1, and 2 may represent smooth muscle cells, endothelial cells, and immune cells, respectively. Interestingly, cluster 6 cells scored high for both atrial cardiomyocytes and fibroblasts. Importantly, a previous murine lineage tracing study determined that c-kit+ cells are primarily endothelial cell progenitors, minimally contribute to cardiomyocytes, and have some propensity to generate fibroblasts, smooth muscle cells, and immune cells [50]. Additionally, lineage tracing studies in various animal models have posited that fibroblasts may be endogenously activated and may be endocardium- or endothelial-derived [51,52,53]. Given our transcriptomic results suggest cluster 6 is a fibroblast-like cell cluster, additional experiments to confirm cell identification and origin are warranted. For now, we subclustered our cluster 6 cells for comparison to previously published fibroblast subpopulations (Figure S5). Notably, single-cell studies in zebrafish [52] and mice [54] have identified anti-Wnt signaling in activated fibroblast subpopulations. While our results suggest some cluster 6 cells express canonical myofibroblast markers (*POSTN*, *PLOD2*, *ACTA2*) and Wnt markers (*WNT5A*, *DKK3*), these results are limited by our cluster 6 cell count.

Finally, we acknowledge the limitations of this study’s design and subsequent results. First, CSCs were passaged several times before performing single-cell sequencing. CSCs constitute a small percentage of cells in the myocardium, and their therapeutic use requires cell expansion [16,26]. Here, we have limited our analyses to CSCs ≤ passage 15 to limit transcriptomic drift and validated c-kit+ expression with flow cytometry after cell expansion (Figure S1). Additionally, we do not discount the potential effect of disease diagnosis on cell composition. CSCs were isolated from cardiac biopsies of pediatric patients undergoing routine cardiac surgery. Considering their source, age and disease variables are inherently dependent. In this study, neonate cells were sourced from patients with critical defects (hypoplastic left heart syndrome, total anomalous pulmonary venous return, and coarctation of the aorta), whereas child cells were isolated primarily from patients with more simple defects (subaortic stenosis, atrial and ventricular septal defects). Future studies including more congenital heart disease patients will help distinguish between age-dependent factors and factors related to a specific disease state.

Overall, we have identified a fibroblast-like population of CSCs, which may drive the suboptimal performance of CSCs derived from older patients. The identification of subpopulations driving (or hindering) therapeutic success will be important for optimizing cell therapy and limiting cell variability.

## Data Availability

The sequencing data reported here have been uploaded to the GEO Database (GSE204928).

## Supplementary Materials

The following supporting information can be downloaded at: https://www.mdpi.com/article/10.3390/jcdd9110374/s1, Figure S1. Characterization of CSCs. Figure S2. Differential expression of genes in cell clusters. Figure S3. Proliferation of VCAN+/ITGA2+ cCSCs and unsorted nCSCs and cCSCs. Figure S4. Pathway analysis of enriched genes from age group regression. Figure S5. Sub-clustering and marker identification of Cluster 6 fibroblast-like cells. Table S1. Patient characteristics (age, disease, cell passage). Table S2. Sequencing metrics for each sample. Table S3. Cell type predictions for each cluster and gene marker dataset. Table S4. Co-expressed gene modules, as determined by Monocle. Table S5. Differentially expressed genes (edgeR); Neonate vs. Child for each cell cluster. Fold-change: Neonate/Child. Table S6. Positive cluster marker gene sets for 13 cell clusters. Markers computed on non-batch corrected data using Seurat’s FindAllMarkers function and Wilcoxon rank sum method. Table S7. Metascape pathway analysis of positive cluster markers in Table S1. Values reported are −log10 (*p*-value). Table S8. Top 20 genes from each age group using the quasi-poisson regression model implemented in Monocle.
