# Supplementary material for: Single-Cell RNA Sequencing Reveals Distinct Cardiac-Derived Stromal Cell Subpopulations
Source: J Cardiovasc Dev Dis. 2022 Nov 1;9(11):374. doi: 10.3390/jcdd9110374 (PMC9693599; doi:10.3390/jcdd9110374)
Supplement: Supplementary file 1 [file jcdd-09-00374-s001.zip › jcdd-1980443-supplementary.pdf]

Supplemental Figures

Title: Single-Cell RNA Sequencing Reveals Distinct Cardiac-Derived Stromal Cell Subpopulations.
Short title: Single cell RNA-sequencing of Pediatric CSCs

Authors: Jessica R. Hoffman*[1,2], Arun R. Jayaraman*[1], Sruti Bheri[1], Michael E. Davis[1,2,3]
*Authors contributed equally
Correspondence: Michael E. Davis, PhD, Biomedical Engineering, Wallace H. Coulter Department of Biomedical Engineering, Georgia Institute of Technology & Emory University School of Medicine, 2015 Uppergate Drive, Rm 346, Atlanta, Georgia 30322, USA. Email: michael.davis@bme.gatech.edu

Affiliations:
1. Wallace H. Coulter Department of Biomedical Engineering, Georgia Institute of Technology & Emory University School of Medicine, Atlanta, GA 30322, USA.
2. Molecular & Systems Pharmacology Graduate Training Program, Graduate Division of Biological & Biomedical Sciences, Laney Graduate School, Emory University, Atlanta, GA 30322, USA.
3. Children's Heart Research & Outcomes (HeRO) Center, Children's Healthcare of Atlanta & Emory University, Atlanta, GA 30322, USA.

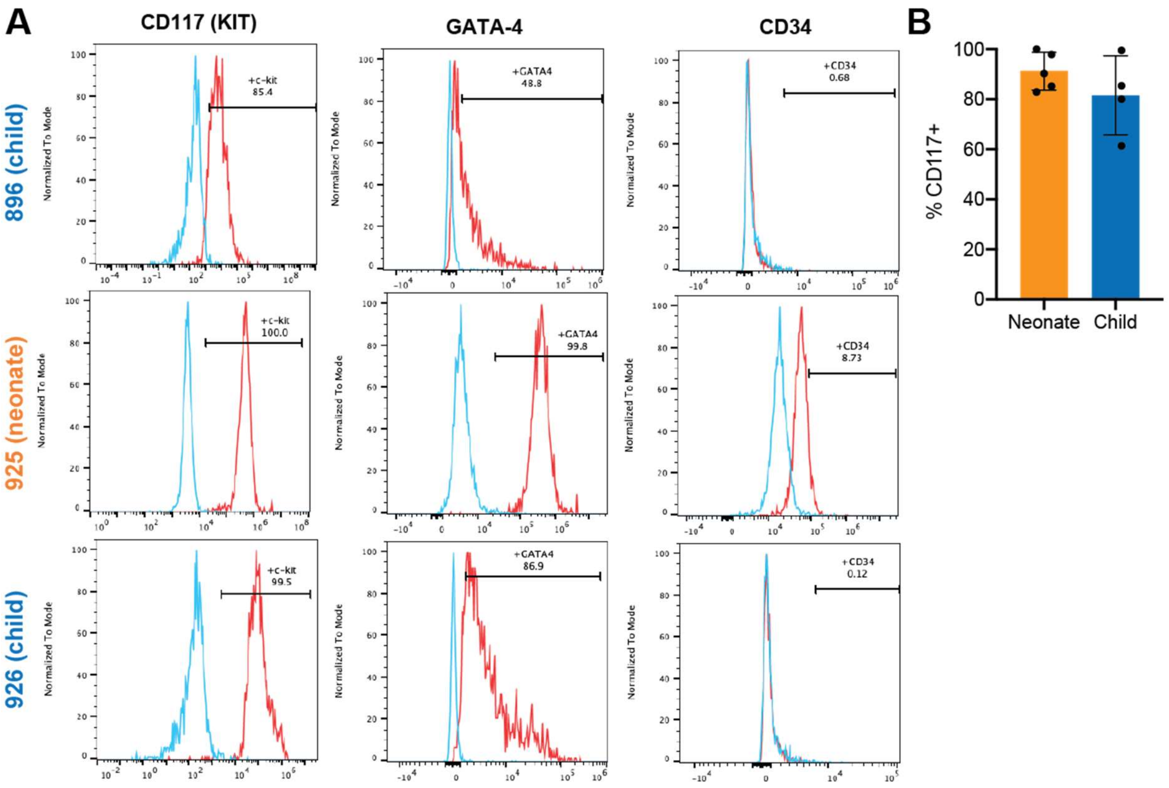

Figure S1. Characterization of CSCs.

(a) Representative histograms of flowcytometry analysis for phenotypic characterization of neonate patient 925 and child patients 896 and 926. CSC populations were positive for c-kit (CD117) and GATA-4 transcription factor, as well as negative for CD34 hematopoietic marker. Blue: unstained control, Red: stained. (b) Percentage of c-kit+ cells in neonate samples (Patients 903, 925, 930, 985, 2016) and child samples (896, 926, 938, 1048). The following antibodies were used at manufacturers' suggested concentration: CD117 (Santa Cruz, sc-5535), GATA-4 (Santa Cruz, sc-9053), CD34 (eBioscience, 11-0341-81). Appropriate secondary antibodies were used after CD117 and GATA-4 primary staining. For GATA-4 staining, cells were permeabilized with 0.1% TritonX-100.

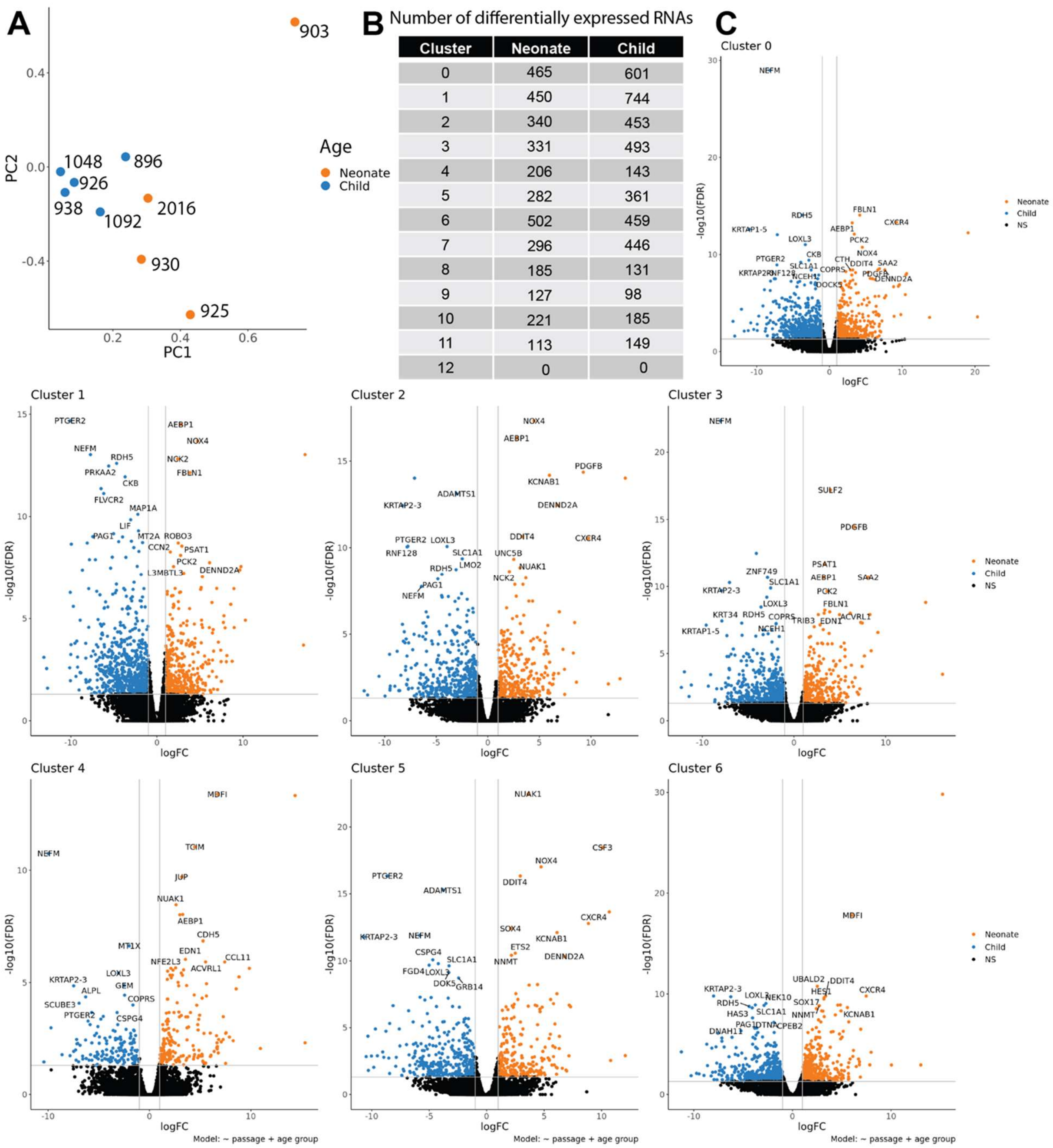

Cluster	Neonate	Child
0	465	601
1	450	744
2	340	453
3	331	493
4	206	143
5	282	361
6	502	459
7	296	446
8	185	131
9	127	98
10	221	185
11	113	149
12	0	0

Figure S2. Differential expression of genes in cell clusters.
(a) Principal component analysis performed after single cell feature expression aggregation by patient ID. (b) Differential expression analysis was performed in edgeR after filtering out lowly expressed genes (filterByExpr, default parameters). The co-variates cell passage and age group were used with a glm fit with Benjamini-Hochberg correction. The number of differentially expressed genes upregulated in neonate and child age groups are listed in the table (FDR < 0.05, log2FC >1). (c) Volcano plots highlighting genes upregulated in neonate cells (orange) and child cells (blue). Volcano plots are shown for cluster 0-6.

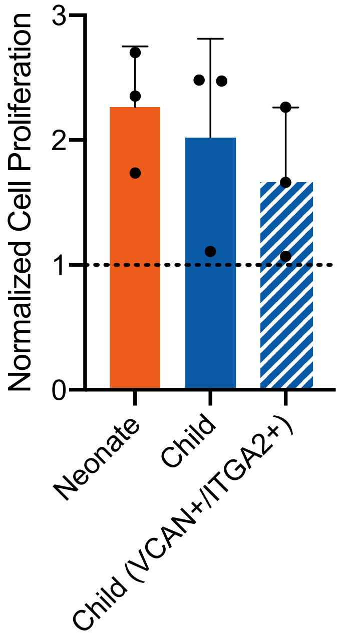

Figure S3. Proliferation of VCAN+/ITGA2+ cCSCs and unsorted nCSCs and cCSCs. CSC proliferation after 48 hrs was measured for nCSCs (patient 2016), as well as unsorted and VCAN+/ITGA2+ sorted cCSCs (patient 926 and pooled CSCs of patients 926, 938, and 902). Proliferation was assessed with the Click-iT^{TM} EdU assay (ThermoFisher Scientific, C10499). Briefly, cells were seeded at 8,000 per well, incubated with EdU for 48 hours, processed as per manufacturer's protocol and then fluorescence (Ex: 568, Em: 585) was quantified with a plate reader (Biotek Synergy 2). Cell proliferation was normalized to corresponding quiesced control group, completed in triplicate, performed three times.

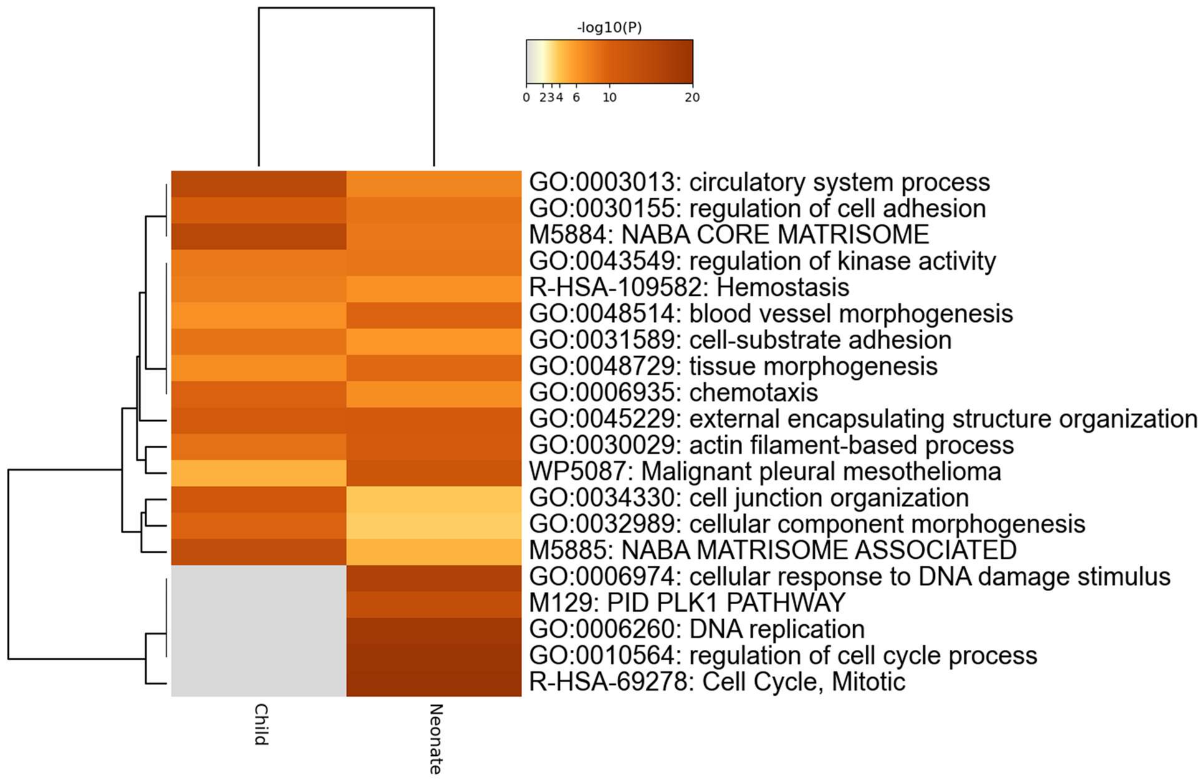

Figure S4. Pathway analysis of enriched genes from age group regression.
A quasi-poisson regression model was built using Monocle to assess gene expression variability due to patient age. Genes with a q-value < 0.05 were input into Metascape to identify enriched biological pathways.

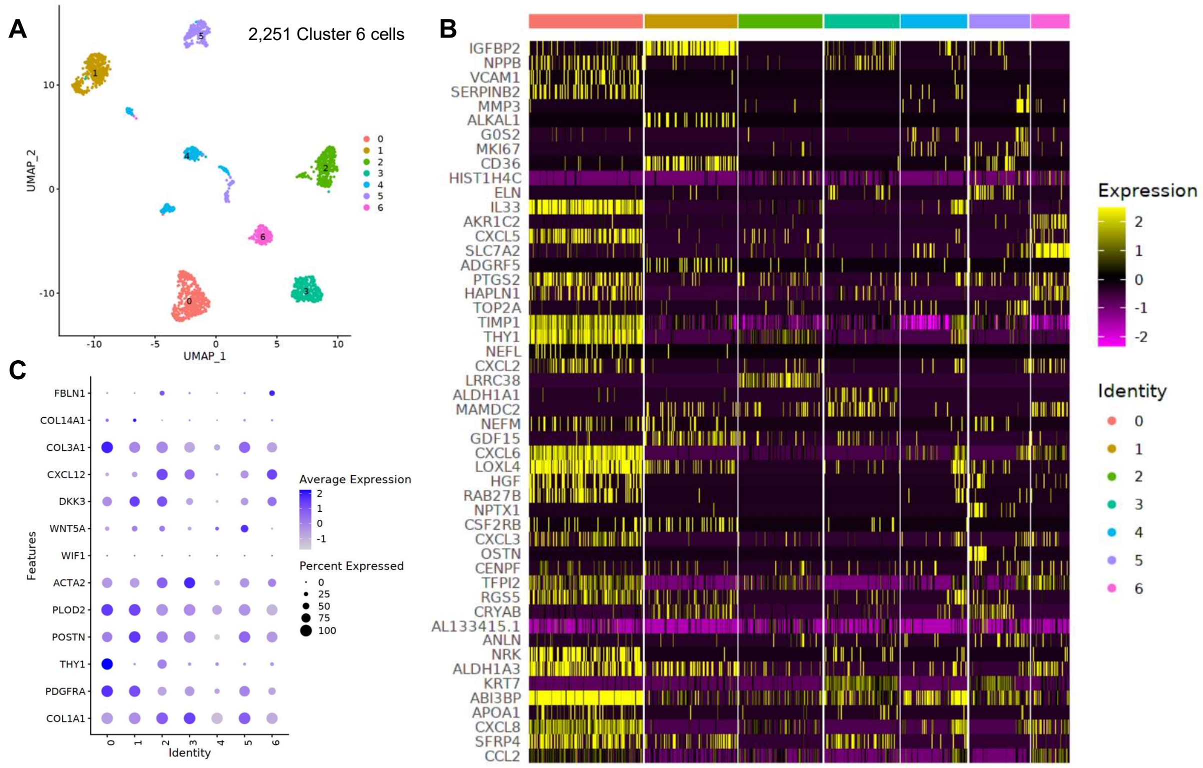

Figure S5. Sub-clustering and marker identification of Cluster 6 fibroblast-like cells.
(a) UMAP and clustering of 2,251 Cluster 6 cells. Dimension reduction and cell clustering was performed with Cluster 6 cells in the same manner as before (methods). (b) Heatmap of the top 50 variable genes across each sub-cluster. (c) Dotplot of selected canonical fibroblast (*COL1A1*, *PDGFRA*), myofibroblast (*POSTN*, *PLOD2*, *ACTA2*), and Wnt-signaling genes identified in activated fibroblast subpopulations (Farbehi et al.).
